# Molecular Engineering of Polymyxin B for Imaging and Treatment of Bacterial Infections

**DOI:** 10.3389/fchem.2021.809584

**Published:** 2022-01-07

**Authors:** Minghao Wu, Shipeng He, Hua Tang, Honggang Hu, Yejiao Shi

**Affiliations:** ^1^ Institute of Translation Medicine, Shanghai University, Shanghai, China; ^2^ School of Engineering and Materials Science, Queen Mary University of London, London, United Kingdom

**Keywords:** polymyxin, bacterial infection, molecular imaging, optical imaging, photodynamic therapy, drug delivery, sustained release, molecular engineering

## Abstract

The emergence of multi-drug resistant bacteria and the lack of novel antibiotics to combat them have led to the revival of polymyxin B, a previously abandoned antibiotic due to its potential nephrotoxicity and neurotoxicity. To facilitate its widely clinical applications, increasing effort has been devoted to molecularly engineer polymyxin B for the targeted imaging and effective treatment of bacterial infections. Herein, the molecular engineering strategies will be summarized in this mini review, with selected recent advances for illustration. Perspective of the challenges and trends in this exciting and eagerly anticipated research area will also be provided in the end. We hope this mini review will inspire researchers from diverse fields to bring forward the next wave of exploiting molecular engineering approaches to propel the “old” polymyxin B to “new” clinical significance in combating bacterial infections.

## Introduction

Driven by the rapid emergence of bacterial resistance throughout the world, tremendous efforts have been made to discover novel antibacterial agents and therapies for the imaging and treatment of bacterial infections. However, given the slow progress in the current antibiotic development pipeline, revitalizing the “old” antibiotics by molecularly engineering them into more effective and safer therapies has been considered as a promising alternative for immediate clinical applications (Årdal et al., 2020).

Among all the “old” antibiotics, the revival of previously abandoned polymyxin B (PMB) is the most striking example. PMB is a family of cyclic non-ribosomal lipopeptides that naturally produced by the Gram-positive *Paenibacillus polymyxa* (Storm et al., 1977). These peptides can selectively bind to the lipopolysaccharide (LPS) on the outer membrane of the Gram-negative bacteria and exert their antibacterial activities by disrupting both the outer and inner membranes of Gram-negative bacteria (Nation et al., 2015). However, due to their reported nephrotoxicity and neurotoxicity, the clinical use of PMB has been discarded (Dai et al., 2019; Oliota et al., 2019). Only in the late 1980s, the PMB was reintroduced as the last-resort for the treatment of infections caused by Gram-negative bacteria that resistant to conventional antibiotics (Velkov et al., 2010). Simultaneously, increasing studies have sought to rationally engineer the PMB molecules to fully unlock their therapeutic potentials.

**FIGURE 1:**
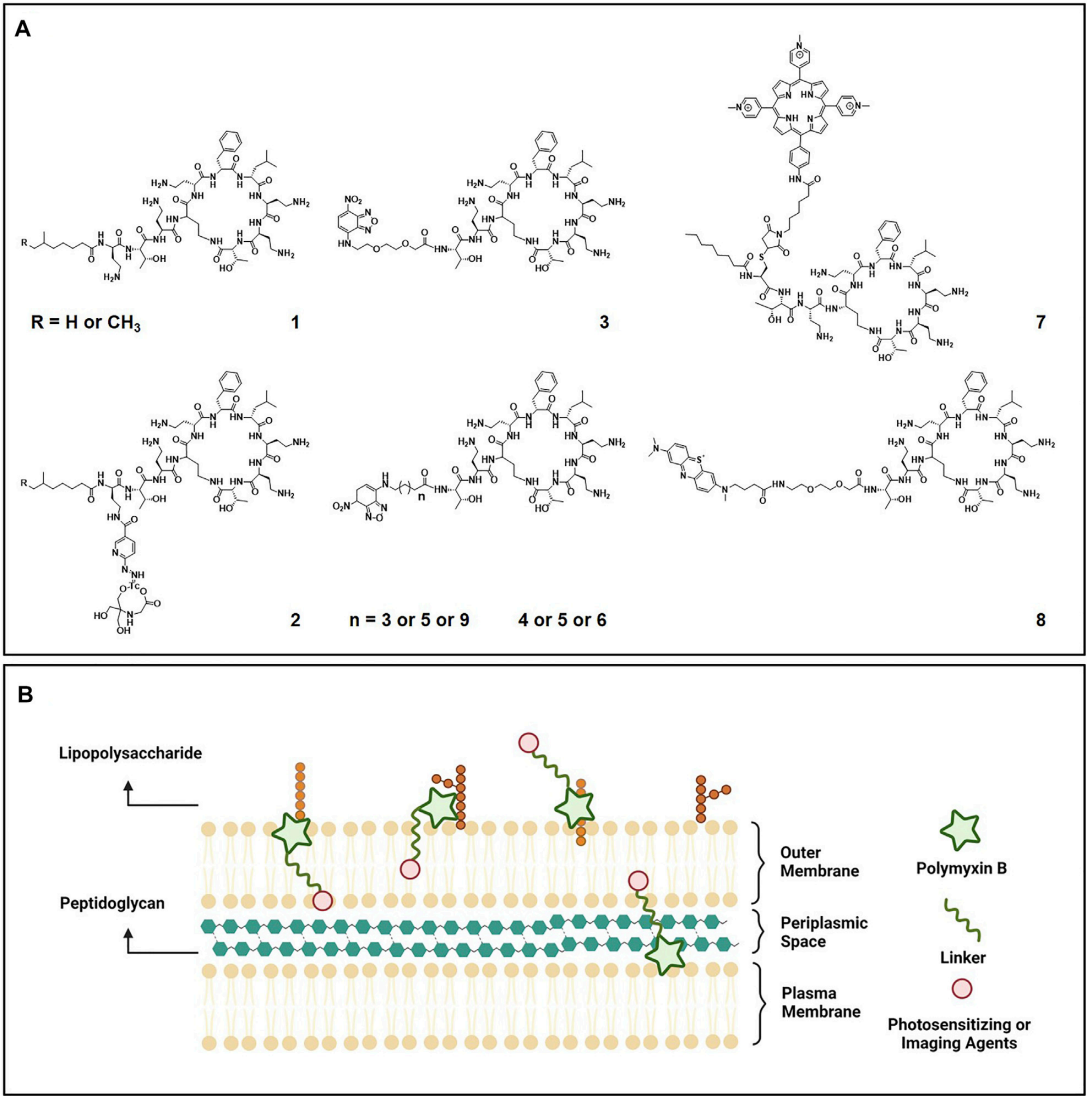
**(A)** Chemical structures of polymyxin B (compound 1) and its engineered molecular imaging agent ^99m^Tc-HYNIC-PMB (compound 2), optical imaging agent NBD-PMB (compound 3-6), and photosensitizing agents (compound 7 and compound 8). **(B)** Illustration of the polymyxin B based imaging and photosensitizing agents targeting Gram-negative bacteria.

An understanding on the activities of PMB require knowledge of its structural properties. The PMB molecule composes of a cyclic heptapeptide headgroup and a linear tripeptide lipid tail. The commercially available PMB, which is produced by fermentation, is a mixture of two main components with closely related structures (compound **1**, Figure 1A). There are five cationic diaminobutyric acid (Dab) residues in PMB, allowing for its accumulation at the anionic bacterial membrane. The specific electrostatic interactions between the positively charged PMB Dab residues and the negatively charged Gram-negative bacterial lipid A phosphates, competitively replace the membrane stabilizing divalent cations and bring the *N*-terminal fatty acyl tail of PMB into proximity with the outer membrane (OM) of Gram-negative bacteria (Clausell et al., 2007). Subsequently, both the hydrophobic lipid tail and _D_-Phe^6^-_L_-Leu^7^ domain of PMB insert into the OM, weakening the packing of adjacent lipid A fatty acyl chain and expanding the monolayer of OM. The permeation of OM enables the PMB to penetrate into the periplasmic space and disrupt the physical integrity of the inner membrane by straddling and exchanging the phospholipid bilayer (Rabanal and Cajal, 2017). The osmotic balance of Gram-negative bacteria is thereby broken, leading to the lytic cell death (Yu et al., 2015).

To improve the microbiological, pharmacological and toxicological profiles of PMB, substantial efforts have been made to discover of PMB analogues based on medical chemistry strategies (Velkov et al., 2010). Structure-activity relationships (SAR) drawn from the antibacterial activities of these analogues indicate that the unique structural properties of PMB is crucial for its targeted LPS binding and effective bactericidal action. For example, substitution of the Dab residues in the cyclic peptide ring of PMB with neutral aminobutyric acid residues resulted in a complete loss of antimicrobial activity (Vaara et al., 2008). While significantly reduced affinity for the isolated rat kidney brush border membranes was observed for these analogues with decreased positive charges, suggesting reduced nephrotoxicity. Proteolytic removal of the *N*-terminal fatty acyl tail and Dab segment generates the polymyxin nonapeptide PMBN. Even though the PMBN lacks direct bactericidal activity, it retains the highly specific LPS binding ability and displays a markedly reduced toxicity profile (Vaara and Vaara, 1983). Based on these collective knowledges on SAR, PMB has been increasingly utilized as functional building scaffold to incorporate additional functional domains, such as imaging and photosensitizing moieties, for the development of advanced therapies for bacterial infections.

In this context, we will summarize the recent advances in exploiting the PMB as building block to design functional molecules or materials for targeted imaging and efficient treatment of bacterial infections. Rather than being comprehensive, we aim to outline the molecular design strategies, illustrated with selected examples, and highlight how the modification and organization of the PMB molecules alter their specificity, potency and clinical relevance towards their widespread adoption to fight bacterial infections. We hope this mini review will provide scientists with diverse molecular engineering approaches, which may represent the next wave of research against bacterial infections, to propel the “old” polymyxin B to “new” clinical significance.

## Imaging of Bacterial Infections

In the clinic, accurate and rapid diagnosis of bacterial infections can aid clinicians in deciding the optimal route and monitoring the effectiveness of treatment. However, conventional identification methods of bacteria, such as culture and colony counting, are labor-intensive and time-consuming (Huang et al., 2021). Although the emerging molecular techniques, including polymerase chain reaction (PCR), enzyme-linked immunosorbent assay (ELISA), chromatography and mass spectrometry, as well as electrochemical sensing, reduce the detection time and improve the detection sensitivity, expensive equipment, professional technicians and complicated operations are required (Tsalik et al., 2018). Besides, these molecular diagnostic methods require clinical samples (e.g., blood, urine, stool etc.), which may not represent the local biology of the exact infection sites (Ordonez et al., 2019). It is therefore imperative to develop novel approaches for the direct imaging of bacterial infections *in vivo*.

Currently, the *in vivo* imaging of bacterial infections remains challenging. Clinically available imaging tools such as radiography, ultrasonography, computed tomography (CT), and magnetic resonance imaging (MRI) function on visualizing the anatomical changes. Consequently, only late-stage bacterial infections, which become systemic and caused significant damage to key tissues and organs, can be diagnosed by these structural imaging approaches (Trampuz and Zimmerli, 2006; Jain, 2017). To make the treatment more effective and less costly, imaging of the early-stage bacterial infections will be essential. Fortunately, both molecular imaging and optical imaging have provided attractive tools to achieve it. However, these two functional imaging techniques are still restricted for their clinical implementation, since they reflect the physiological changes during all the inflammatory process, cannot reliably distinguish bacterial infections from inflammations caused by other diseases (Ordonez et al., 2019).

To realize the full potential of these functional imaging techniques for more specific detection of bacterial infections, substantial efforts have been devoted to develop the bacteria-targeted imaging strategies. Since bacterial (prokaryotic) cells are evolutionarily and phylogenetically distinct from mammalian (eukaryotic) cells in proteins, nucleic acids, cell wall components and even metabolism, targeting molecules including antibodies, DNA/RNA ligands, antibiotics and also metabolizable compounds have been utilized correspondingly to facilitate the targeted bacterial infection imaging (van Oosten et al., 2015; Mills et al., 2016). For example, maltodextrin (e.g. maltose, maltotriose, and maltohexose) is the major source of glucose for bacteria and is taken up by bacteria through the maltodextrin transporter, which is not expressed in mammalian cells (Dahl and Manson, 1985). Accordingly, several maltodextrin-based imaging probes have been exploited as metabolic substrates for imaging of bacterial infections with high specificity over sterile inflammations (Ning et al., 2011; Zlitni et al., 2020).

Additionally, infections caused by specific species of bacteria can also be differentially imaged with the help of the bacterial selectively interacted molecules. Since the efficacy spectrum of most antibiotic is dependent on the species of bacteria, labelled antibiotics have been extensive studied for the bacteria-specific imaging. Best known antibiotic being exploited is vancomycin, a glycopeptide that binds efficiently to the *D*-ala-*D*-ala moiety of lipid II in the nascent cell wall of Gram-positive bacteria (Pasquina-Lemonche et al., 2020). Promising results were reported on the use of NIR fluorophore IRDye 800 CW conjugated vancomycin (vanco-800CW) for real-time *in vivo* imaging of Gram-positive bacterial infections on both murine myositis and human post-mortem implant models (Van Oosten et al., 2013). As for imaging of Gram-negative bacterial infections, the cationic and cyclic antibiotic PMB that selectively binding to lipid A of LPS on the outer membrane of Gram-negative bacteria have been increasingly exploited (Figure 1B).

By radiolabeling PMB with ^99m^Tc *via* a succinimidyl-6-hydrazinonicotinate hydrochloride (HYNIC) linker, ^99m^Tc-HYNIC-PMB (compound **2**, Figure 1A) was obtained most recently as a new single photon emission imaging agent for the non-invasive identification of Gram-negative bacterial infections (Auletta et al., 2021). In the mice with right thigh bacterial infections, the lateral tail vein injected ^99m^Tc-HYNIC-PMB exhibited significantly higher target-to-background ratio for Gram-negative bacteria than Gram-positive bacteria under gamma camera imaging. However, the HYNIC react with PMB mainly via the *γ*-amine groups of α, *γ*-diaminobutyric acid, which are also responsible for the specific binding of LPS in Gram-negative bacteria. Hence, investigations are still needed to deeply understand the mechanism of action and behavior of ^99m^Tc-HYNIC-PMB.

Besides being exploited as nuclear molecular imaging agents, PMB has also been chemically modified to facilitate the optical imaging of Gram-negative bacterial infection. By removal of the hydrophobic tail and two amino acid residues and replaced with various linkers attached to the fluorophore 7-nitrobenz-2-oxa-1,3-diazole (NBD), a panel of NBD-PMB (compound **3-6**, Figure 1A) were synthesized and evaluated for their Gram selectivity (Akram et al., 2018). Compared to NBD-PMX with progressive lengthening of hydrophobic acyl spacers (compound **4-6**), the NBD-PMX with a hydrophilic amino-PEG2-carboxylate spacer (compound **3**) displayed the highest preference in labeling Gram-negative over Gram-positive bacterial species and mammalian cells. Its mode of action was further demonstrated to be connected with the specific interaction between the cyclic ring of PMB and lipid A of LPS, since it displayed a lack of binding to the K56-2 bacterial strain that mutated in lipid A expression. After intrapulmonary micro-dosing, the NBD-PMX probe (compound **3**) enabled *in situ* identification of Gram-negative bacteria in distal human airways and alveoli within minutes by optical endomicroscopy, representing potentially significant advantageous over current technologies.

Since the aforementioned two PMB based imaging agents are administrated by either lateral tail vein injection or intrapulmonary micro-dosing, the existing of gut bacteria will not introduce any background noise. While being recognized as a newly discovered organ, gut microbiota has also been extensively studied. Emerging imaging strategies for the commensal microbiota and pathogenic bacteria in the gut have been recently summarized in an inspiring review that is encouraged to read (Lin et al., 2021).

## Treatment of Bacterial Infections

### Polymyxin B Based Photosensitizing Agents

Photodynamic therapy (PDT) is a medical treatment that utilizes a light source to active a non-toxic photosensitizer (PS) in the presence of oxygen, generating highly cytotoxic reactive oxygen species (ROS) and destroying abnormal cells consequently (Castano et al., 2004). It is initially used for the treatment of skin and cancer diseases (Agostinis et al., 2011), while has gained great attention nowadays as a promising alternative to the emerging ineffectiveness of antibiotic treatment for bacterial infections (Wainwright et al., 2017) (Klausen et al., 2020). Compared to the conventional antibiotic treatment, the PDT not only has more rapid and broader spectrum antimicrobial properties, but also be unlikely to give rise to the bacterial resistance, since the produced ROS can damage nearly all types of biomolecules, leading to bacterial death (Vatansever et al., 2013). Despite these outstanding advantageous, two major drawbacks of PS, which lie in its 1) poor selectivity and off-target damage to mammalian cells; 2) low penetrability and reduced efficiency against Gram-negative bacteria and bacteria in biofilms, still limit the clinical adoption of PDT for antibacterial treatments.

To improve the selectivity and penetrability of PS towards pathogenic bacteria, PMB has been utilized as a functional building block to engineer PS conjugates for the targeted antibacterial PDT (Figure 1B). Using a thiol-maleimide “click” coupling, a cationic porphyrin was covalently attached to a cysteine modified PMB derivative (compound **7**, Figure 1A) (Le Guern et al., 2017). Compared to the cationic porphyrin alone, enhanced bactericidal efficiency was achieved with the addition of modified PMB derivative, which could anchor to and weaken the bacterial membranes (Nitzan et al., 1992). However, the modification induced reduction of cationic charges of PMB and rendered its specificity against Gram-negative bacteria, with an emerging activity against Gram-positive *S. aureus* being observed. To eliminate the potential risk of rising PMB resistance, four diaminobutyric acid residues of compound **7** were substituted with the lysine ones in a following study (Le Guern et al., 2018). The lysine analogue of compound **7** showed reduced dark toxicity (bactericidal activity), while maintained the high photoinactive potency against both Gram-positive and Gram-negative bacteria.

In contrast to the above studies that engineered with the entire PMB molecule, Ucuncu et al. removed the hydrophobic tail of PMB and conjugated it with an FDA approved PS methylene blue (MB) through a short polyethylene glycol (PEG) linker (compound **8**, Figure 1A) (Ucuncu et al., 2020). Without the hydrophobic tail, compound **8** exhibited decreased dark toxicity, while preserving the high Gram-negative bacteria specificity. In addition, the comparatively hydrophilicity and small size of compound **8** presumably allow its deeper penetration into the thick extracellular polymer matrix of biofilms and well-organized outer membrane of Gram-negative bacteria. Upon light irradiation, outstanding killing potency was achieved against Gram-negative *E. coli* not only in the planktonic status, but also in the infected porcine skin and most importantly biofilms. Moreover, no side effect of compound **8** was observed on human erythrocytes, demonstrating its high therapeutic potential to treat Gram-negative bacterial infections.

### Polymyxin B Based Delivery Systems

**FIGURE 2:**
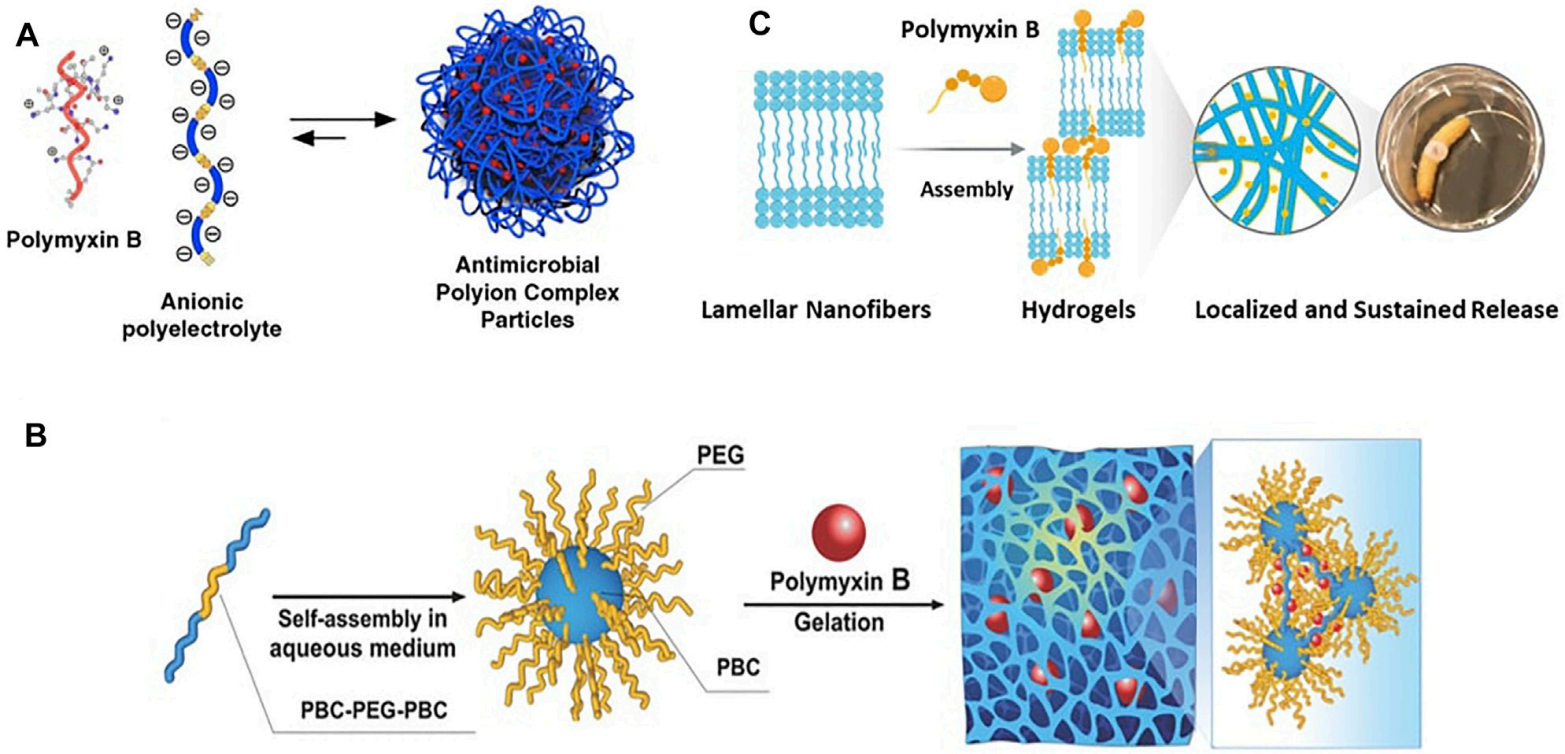
The polymyxin B-based delivery systems: **(A)** Polyion complex nanoparticles co-assembled from polymyxin B with poly(styrene sulphonate). Reproduced from (Insua et al., 2017), published by Elsevier Ltd., **(B)** polymyxin B crosslinked hydrogels based on the core-shell micelles assembled from the phenylboronic acid functionalized polycarbonate and polyethylene glycol triblock copolymer (PBC-PEG-PBC). Adapted with permission from (Obuobi et al., 2018). Copyright 2018, Wiley-VCH GmbH, **(C)** polymyxin B crosslinked hydrogels based on the lamellar nanofibers assembled from rationally designed peptide amphiphiles. Adapted from (Shi et al., 2021), published by Wiley-VCH GmbH.

Currently, the major factor limiting the wide applications of PMB in clinical practice is its potential nephrotoxicity and neurotoxicity, which are considered to be dose-dependent (Dai et al., 2019; Oliota et al., 2019). Therefore, a targeted or localized delivery system is critically needed to reduce the dose of administration and consequently toxicity of PMB. Fortunately, the thriving field of nanomedicine over the past few decades has provided advantageous possibilities to deliver drugs to their site of action and maximize their therapeutic outcomes. Diverse delivery systems based on nanoparticles, liposomes, hydrogels, micromembranes and microneedles have been developed for the targeted and controlled release of PMB (Dubashynskaya and Skorik, 2020). While in most of the nanomaterial systems, such as liposomal formulations, PMB is just physically entrapped inside the preformed delivery vehicles. Considering its chemical structure, which contains five cationic amine residues and a lipophilic acyl tail, PMB has also been increasingly exploited as building block for the construction of its own delivery systems. For instance, the anionic poly(styrene sulphonate) (PSS) was utilized to co-assemble with cationic PMB for the formation of polyion complex (PIC) nanoparticle (Figure 2A) (Insua et al., 2017). By adjusting the ratio of PSS and PMB, stable colloidal PIC nanoparticles with an average particle diameter of 140 ± 30 nm were prepared and demonstrated to preserve inhibition activity against *Pseudomonas aeruginosa* for at least 8 h. The inhibition time of bacterial growth was further proven to be formulation-dependent, based on a competing balance between the content of PMB and stability of the co-assembled PIC nanoparticles.

In addition to nanoparticles, PMB has also been utilized as crosslinker for the formation of hydrogels. For example, phenylboronic acid (PBA) functionalized polycarbonate (PBC) and polyethylene glycol (PEG) triblock copolymer (PBC-PEG-PBC) was synthesized and reported to self-assembled into core-shell micelles in aqueous solution (Obuobi et al., 2018). Upon addition of PMB, hydrogel formation was achieved *via* the hydrogen bond, hydrophobic interactions and electrostatic forces between the functional PBA blocks of copolymer and the amine groups of PMB (Figure 2B). By repositioning the substituents on the aryl ring of PBA, hydrogels with varying swelling behaviors, mechanical strengths, as well as PMB loading and release capacities could be obtained. The optimum *meta*-PBA hydrogel exhibited sustained PMB release over 48 h, which further enabled the prolonged antibacterial activity of PMB against *Pseudomonas aeruginosa* burn wound infections in mouse model. In another example most recently reported by us, peptide amphiphile composed of a glutamic acid–threonine–glutamic acid head and *N-*terminal acryl tail was rationally designed and self-assembled into lamellar nanofibers in aqueous solution (Shi et al., 2021). With PMB bearing the opposite charges as ionic cross-linker, the lamellar nanofibers were triggered to form robust hydrogels with tunable mechanical properties (Figure 2C). Sustained PMB release *via* diffusion-dependent kinetics over a 5-day period was observed from the hydrogels, which were demonstrated to be effective for the treatment of *Pseudomonas aeruginosa* infection in the *Galleria mellonella* burn wound infection model. Moreover, with the successful incorporation of additional fusidic acid, the porous hydrogels displayed complementary antibacterial effect against Gram-positive bacteria and also enhanced antibacterial effect against Gram-negative bacteria, envisioning their potential for combined antimicrobial therapy. However, *in vivo* evaluations of the toxicological profiles of PMB (Chai et al., 2020), in terms of nephrotoxicity and neurotoxicity, are lacking in all the presented delivery systems and are needed to be investigated in the future.

## Conclusion and Outlook

As outlined in this mini review, revitalizing the “old” PMB for “new” clinical significance in the imaging and treatment of bacterial infections is still in its infancy, but emerging progresses have been made based on the molecular engineering approach. Being exploited as a functional scaffold, the PMB molecule has been rationally engineered into diverse molecules and materials to achieve the targeted imaging and efficient treatment of bacterial infections.

Considering its specific interaction with the lipid A of LPS on the outer membrane of Gram-negative bacteria, PMB has been engineered not only as imaging agents to facilitate the targeted molecular imaging and optical imaging of Gram-negative bacterial infections, but also as photosensitizing agents to enable the targeted PDT for the effective treatment of Gram-negative bacterial infections. Since PMB anchors to bacterial membranes mainly *via* the electrostatic interactions rather than any specific receptors, it relatively rare to induce bacterial resistance compared to other antibiotics. However, with its increasing clinical use, polymyxin resistance is also appearing at an alarming rate. Bacterial surfaces of the resistant strains were observed with reduced negative charges due to the controlled addition of positively charged residues, such as 4-amino- *l* -arabinose, phosphoethanolamine and/or galactosamine to LPS (Moffatt et al., 2019). As a consequence, the specific interaction between polymyxin and LPS is reduced. Therefore, despite the promising progress made on the engineering of PMB based imaging agents and photosensitizing agents, strategies and approached aimed at decreasing the antibacterial activity of the peptide moiety while preserving its target specificity are still needed in the future. Furthermore, PMB based agents with both imaging and photosensitizing capacities should be rationally engineered so that the targeted theranostic of bacterial infections would be achieved.

As for the molecularly engineered delivery systems, the release of PMB in a more controlled manner should be considered. Current systems are mainly co-assembled based on the non-covalent interactions between PMB and other functional building blocks, thus the release of PMB is diffusion-depended with burst release being typically observed. To reduce the dose related toxicity and prolong the antibacterial efficiency of PMB, the specific conditions of bacterial infected environment, such as pH gradient, overexpression of certain enzymes and ROS storage, should be utilized to rationally design self-assembling PMB conjugates, which could form supramolecular delivery systems with tunable linear release rate (Chakroun et al., 2019). Additionally, complementary agents with different antibacterial mechanism could be co-delivered to achieve synergistic antibacterial effect for the combined antimicrobial therapy. Meanwhile, besides the hydrogel formulation engineered for topical administration of PMB, other nanomaterial delivery systems should also be developed to facilitate the oral, inhalable and transdermal administrations. Despite the above challenges, it is conceivable that combined effort by researchers from diverse field would bring forward a spectrum of molecular engineering approaches to maximize the therapeutic potentials of polymyxin B for combating bacterial infections.
